# Addition of silver nanoparticle solutions to alginate: microbiological and mechanical analysis

**DOI:** 10.1590/0103-644020266966

**Published:** 2026-08-07

**Authors:** Larissa Paulino de Figueiredo, Analia Gabriella Borges Ferraz Facury, Eduardo Martinelli Franco, Gilberto Antonio Borges, Roberto Nascimento Silva, Lourenço Correr-Sobrinho, José Guilherme Neves, Ana Rosa Costa

**Affiliations:** 1 Department of Orthodontics, Hermínio Ometto Foundation - FHO, Araras, SP, Brazil; 2 Department of Restorative Dentistry, Dental Materials area, Piracicaba Dental School, State University of Campinas, FOP - UNICAMP, Piracicaba, SP, Brazil; 3 Department of Microbiology, Piracicaba Dental School, State University of Campinas, FOP - UNICAMP, Piracicaba, SP, Brazil; 4 Department of Restorative Dentistry, Universidade de Uberaba - UNIUBE, Uberaba, MG, Brazil; 5 Department of Biochemistry and Immunology, Faculty of Medicine of Ribeirão Preto - USP, Ribeirão Preto, SP, Brazil

**Keywords:** Alginate, Chlorhexidine, Antibacterial Agents, Streptococcus mutans, Candida albicans

## Abstract

This study assesses the antimicrobial activity and physical alterations of alginate impression material in the presence of silver nanoparticle (AgNP) solutions. The samples were assigned to five groups according to the type of alginate and solution: (JW) Jeltrate + distilled water; (JCHX) Jeltrate + 0.2% chlorhexidine digluconate; (JAgNP_0.2%) Jeltrate + 0.2% AgNP; (JAgNP_1%) Jeltrate + 1% AgNP; and (AW) Avagel + distilled water. AgNPs were synthesized from the fungus *Trichoderma reesei*. Fifty impressions were made (n=10) from a matrix, from which plaster models were fabricated. Surface detail reproduction and dimensional stability were analyzed under a light microscope. Surface detail reproduction was achieved by replicating a 50µm line on the plaster model. Dimensional stability was calculated from measurements of X’ and X’’ in the matrix and plaster models. The antimicrobial effect was assessed independently by measuring the formation of zones of inhibition around the alginate molds (n=3) in agar medium containing *Streptococcus mutans* and *Candida albicans*. Dimensional stability values were subjected to the ANOVA and Tukey’s post-hoc test (α=0.05). JCHX showed the highest dimensional stability and differed significantly from the other groups (p<0.05). A 50µm line was reproduced in the plaster for all tested groups, regardless of the impression material and antimicrobial solution. AgNPs incorporated into the alginate mixture did not produce dimensional changes in the alginate mold. AW and JCHX exhibited antimicrobial potential, as evidenced by bacterial inhibition. Jeltrate or Avagel, regardless of AgNP concentration, did not demonstrate antimicrobial activity.

## Resumo

Este estudo avaliou a atividade antimicrobiana e as alterações físicas do material de moldagem alginato com soluções de nanopartículas de prata (NPAg). As amostras foram distribuídas em cinco grupos de acordo com o tipo de alginato e solução: (JA) Jeltrate + água destilada; (JCHX) Jeltrate + digluconato de clorexidina a 0,2%; (J NPAg _0,2%) Jeltrate + NPAg a 0,2%; (J NPAg _1%) Jeltrate + 1% NPAg e (AA) Avagel + água destilada. NPAg foram sintetizadas a partir do fungo *Trichoderma reesei*. Cinquenta impressões foram realizadas (n=10) de uma matriz metálica, a partir da qual foram confeccionados modelos de gesso. A reprodução dos detalhes da superfície e a estabilidade dimensional foram analisadas através da microscópia óptica. A reprodução de detalhes da superfície foi obtida pela replicação da linha de 50 μm no modelo de gesso. A estabilidade dimensional foi calculada pelas medidas entre X' e X'' da matriz e do modelo de gesso. O efeito antimicrobiano foi avaliado de forma independente com base na formação de zonas de inibição entre os moldes de alginato (n=3) e o meio de ágar contendo *Streptococcus mutans* e *Candida albicans*. Os valores de estabilidade dimensional foram submetidos à ANOVA e ao teste post-hoc de Tukey (α= 0,05). JCHX apresentou maior estabilidade dimensional e apresentou diferenças significativas em relação aos demais grupos (p<0,05). A linha de 50 μm foi reproduzida no gesso para todos os grupos testados, independentemente do material de moldagem e da solução antimicrobiana. NPAg incorporadas na mistura de alginato não produziram alterações dimensionais no molde de alginato. AA e JCHX exibiram potencial antimicrobiano, evidenciado pela inibição bacteriana. Jeltrate ou Avagel, independentemente da concentração da solução de nanopartículas de prata, não demonstraram atividade antimicrobiana.

**Figure:**
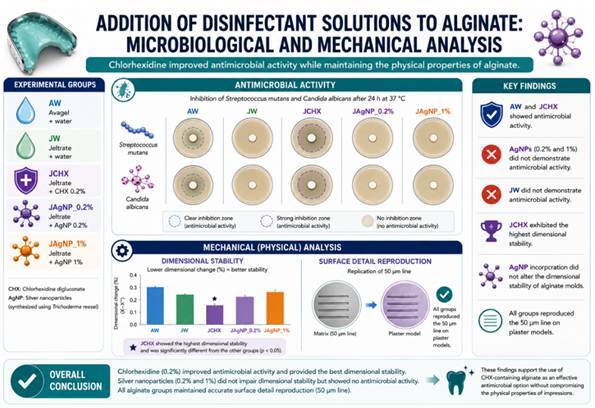

## Introduction

Dental impressions are a recurrent procedure in dental clinical practice, and diverse molding materials, such as alginate, are available on the market ^1^. Alginate is an irreversible hydrocolloid material widely used for the fabrication of dental models ^2^. Despite the increasing adoption of digital workflows, conventional impression materials such as alginate remain widely used ^3^ ^,^ ^4^ in many clinical and educational settings, and their disinfection remains an important aspect of infection control in dentistry. After the mold is removed from the patient’s oral cavity, it is often contaminated with microorganisms due to direct contact with oral fluids, such as saliva and blood, which serve as vectors for disease transmission ^1^. Despite the increasing adoption of digital workflows and intraoral scanning, alginate impressions remain widely used in public health systems, teaching clinics, and resource-limited environments where digital equipment is not routinely available. Recent studies continue to document microbial contamination in irreversible hydrocolloids and evaluate disinfection methods due to persistent biosafety concerns in daily clinical practice ^1^ ^,^ ^5^ ^,^ ^6^.

Dental molds obtained via the molding process must be chemically disinfected before they are filled with plaster and sent to the prosthetic laboratory to prevent them from acting as vectors for the transmission of microorganisms or infectious diseases ^7^. Disinfection of mold is an effective biosafety measure for dental professionals, such as dental surgeons, dental hygienists, and prosthetists, preventing cross-contamination ^8^.

When it comes to antimicrobial substances for the disinfection of alginate molds, nanotechnology is noteworthy, including nanoscale materials (<100 nm) ^5^ ^,^ ^9^. These nanoparticles can originate from primary sources, i.e., chemically or biologically synthesized ^10^. Silver nanoparticles (AgNPs) have attracted significant attention due to their remarkable properties, including a large surface area and excellent antimicrobial activity ^11^. Biogenically synthesized AgNPs, such as those produced by *Trichoderma reesei*, exhibit distinct surface characteristics, heterogeneous size distributions, and potentially lower cytotoxicity compared to chemically synthesized nanoparticles ^12^. These properties may influence antimicrobial behavior and interaction with hydrocolloid matrices, yet their performance when incorporated into the mixing solution of irreversible hydrocolloids has not been fully investigated. As a result, AgNPs have been applied in the health sector, including dentistry ^11^ ^,^ ^13^ ^,^ ^14^, given that Ag is a chemical element with antimicrobial potential and is considered safe and effective for killing more than 650 disease-causing microorganisms.

The biological synthesis of AgNPs by microorganisms has become an eco-friendly approach ^9^ ^,^ ^15^. It is also a more straightforward, cost-effective alternative with potent bactericidal activity against gram-positive and gram-negative pathogens ^11^. Moreover, it is highly efficient, stable, and nontoxic ^16^, with AgNPs readily soluble in water ^17^.

A previous study incorporated AgNPs into irreversible hydrocolloid impressions as antimicrobial agents without adversely affecting their properties, depending on the material type ^15^. Furthermore, 80-100 nm AgNPs exhibited greater antimicrobial activity than Zelgan Plus (Dentsply) irreversible hydrocolloid in a dose-dependent manner. The addition of 0.5% and 1% by weight did not interfere with the properties of the material. However, higher concentrations (2% and 5%) caused gelation time changes and material strength ^18^ ^,^ ^19^. Conversely, another study ^20^ found that self-disinfection of Lascod type A irreversible hydrocolloid (Florence, Italy) with 0.2% chlorhexidine (CHX) solution or alcohol-free commercial mouthwash had similar results to self-disinfection with incorporation of AgNP into the powder (0.1% and 0.2% with 5-8 nm nanoparticles) against the five investigated microorganisms.

Many studies have evaluated the stability and antimicrobial activity of alginate with different disinfectant solutions ^5^ ^,^ ^15^ ^,^ ^18^ ^,^ ^19^ ^,^ ^20^. However, further studies are needed to assess the effect of 0.2% and 1% biologically synthesized AgNP solutions incorporated into the water during alginate powder manipulation. Although several studies have evaluated the incorporation of disinfectant agents into alginate, little is known about the behavior of biologically synthesized AgNPs mixed into the liquid phase, particularly regarding their stability, dispersion within the hydrocolloid network, and potential antimicrobial effects. Addressing these aspects may help identify alternative self-disinfecting strategies in settings where immersion protocols or digital workflows are not feasible. Therefore, this study aimed to evaluate whether incorporating biogenically synthesized silver nanoparticles into the mixing solution of alginate impression materials affects surface detail reproduction, dimensional stability, and antimicrobial activity. By testing two nanoparticle concentrations and comparing them with chlorhexidine and commercial controls, this study sought to determine whether this approach could represent a feasible self-disinfection strategy without compromising critical physical properties. The following hypotheses were stated: 1) incorporation of AgNP into the mixing solution does not affect surface detail reproduction and dimensional stability of alginate molds, and 2) AgNPs do not inhibit microbial growth.

## Materials and methods

### Experimental design

**Figure 1:**
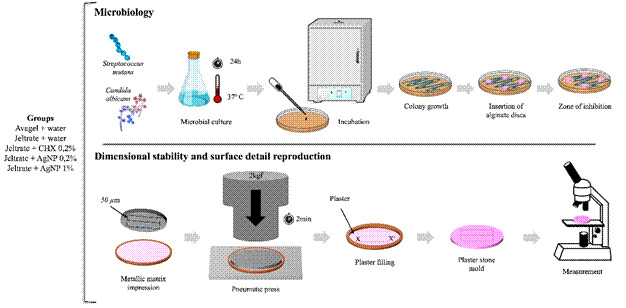
Experimental design

The methodological steps shown in Figure 1 were followed.

### Biological synthesis of AgNPs

The fungus *Trichoderma reesei* was grown on Petri dishes containing MEX-agar medium and incubated in an oven at 28 °C until fungal growth and sporulation occurred within 7 days. The spores were then collected, inoculated into 100 mL of potato dextrose agar, and incubated at 30 °C under low-oxygen conditions in an oven for 7 days. *Trichoderma reesei* produced enzymes capable of generating AgNPs under low oxygen conditions. Silver was biologically synthesized in an amber-colored Erlenmeyer flask by mixing the culture supernatant with aqueous silver nitrate (AgNO_3_) at a concentration of 5 mM (range of 1 to 10 mM). After mixing, the pH was adjusted to 8.5. The mixture was stored in the dark at 40 °C under rotation at 200 rpm for ten days ^21^.

### Microorganisms

*Candida albicans* CBS 562 and *Streptococcus mutans* UA159 were selected based on the microbial composition of oral biofilms and the microorganisms found on dental molds from patients.

### Dimensional stability and surface detail reproduction

Dimensional stability and surface detail reproduction were assessed in accordance with ISO 1563. Five plaster stone models (n = 5) were manufactured for each group according to Guiraldo et al. ^22^. Molds were prepared on a matrix (outer diameter: 38 mm and inner diameter: 29.97 mm) containing three parallel lines measuring 20, 50, and 75 μm in width and 25 mm in length, spaced 2.5 mm apart. Two additional lines, X and X′, were used to determine dimensional stability and surface detail reproduction along the 50 μm line.

Before molding, the matrix was ultrasonically cleaned and dried with compressed air. The irreversible hydrocolloid alginate-based molding materials - Jeltrate Dustless (type II - regular setting) and Avagel, both from Dentsply Sirona, Milford, DE, USA, the latter of which contains CHX, were prepared according to the manufacturer’s instructions.

A perforated metal tray (inner diameter 31 mm, height 5 mm) was placed on a glass plate and filled with the molding material. The tray was joined to the matrix, and a 2 kgf pneumatic press was used to apply pressure and simulate the molding process, allowing the excess material to overflow. The molds were removed 2 min after the material lost its sticky consistency. The molds were rinsed with 150 mL of distilled water and dried afterward.

During the preparation of the alginate powder mixture according to the manufacturer’s instructions, the liquid component was completely replaced by disinfectant solutions. Distilled water was substituted with either 0.2% CHX, prepared at Proderma (Piracicaba, SP, Brazil), or with previously synthesized AgNPs solutions at concentrations of 0.2% and 1.0%. Biogenic AgNPs produced by *Trichoderma reesei* are typically reported to present particle sizes in the nanometric range (approximately 5-50 nm) ^21^. The manufacturer's recommended powder-to-liquid ratio was maintained for all groups.

A single operator manually stirred the mixture for 45 seconds using a spatula and a plastic bowl. The samples were then split into five groups based on the type of disinfection and the disinfectant solution used:

JW: Jeltrate Dustless + distilled water

JCHX: Jeltrate Dustless + 0.2% CHX solution

JAgNP_0.2%: Jeltrate Dustless + 0.2% AgNP solution

JAgNP_1%: Jeltrate Dustless + 1% AgNP solution

AW: Avagel + distilled water

The alginate molds were subsequently rinsed with 150 mL of distilled water, dried, and immediately filled with plaster (Durone IV; Dentsply Caulk), as per the manufacturer's instructions. The plaster stone models were removed from the trays containing the alginate mold 1 hour after the plaster was mixed.

### Light microscopy

Surface detail reproduction measurements were conducted under a light microscope (SZM; Bel Engenharia, MI, Italy) on the plaster stone models at 4x to 12x magnifications to check whether the 50 μm-wide line was accurately replicated along the 25 mm length between the intersection of reference lines (X and X′), in compliance with ISO 1563 standard.

Dimensional stability measurements were conducted on the plaster stone models under a light microscope (STM; Olympus Optical Co Ltd, Japan) with a precision of 0.0005 mm, expressed as a percentage (L) and calculated in accordance with ISO 1563 standard using the equation:

L= [(L2 - L1) / L1] x 100

L1 represents the distance between the lines in the matrix, while L2 denotes the distance between the lines in the plaster stone models.

### Preparation of alginate discs

The powder-to-water ratio in the alginate mold material was adjusted according to the manufacturer’s instructions. The mixture was stirred by a single operator for 45 seconds using a spatula and a plastic bowl. The mixture was then poured into a cylinder (6 mm in diameter and 1 mm in thickness). Excess material was removed by compression with a sterile glass blade. Three discs were prepared for each group and bacterial strain ^23^.

### Antimicrobial activity

Antimicrobial activity was assessed by the disc diffusion method on an agar medium. The discs were placed with sterile tweezers onto a Petri dish containing Mueller-Hinton (MH) agar, which had been previously inoculated with the microorganisms to be tested. The control sample was autoclaved with distilled water. Following incubation, clear zones, or zones of inhibition, are expected to form around the specimens. The following antibiotics were tested: 0.2% CHX and 0.2% and 1% AgNPs. Two microorganisms were used: *S. mutans* UA 159 and *Candida albicans* CBS 562.

The microbial plates and discs were incubated at 35-37 °C for bacteria for 24-48 h and at 25-27 °C for fungi for 48-72 h. The diameter of the inhibition zones was measured directly on the agar surface by a single operator using a millimeter ruler, following the shortest-path criterion ^20^. For each group and microorganism, the mean inhibition zone (in mm) was calculated from the three discs prepared for each condition.

### Statistical analysis

The data were tested for normality and homogeneity. Subsequently, a one-way analysis of variance (ANOVA) was conducted, followed by Tukey’s post hoc test (α = 0.05). The significance level was set at 95%.

## Results

### Dimensional stability

The highest mean dimensional accuracy and the highest statistically significant level of dimensional accuracy were observed with Jeltrate + 0.2% CHX (Table 1).

**Table 1.** Mean values (standard deviation) of dimensional stability (%) for the tested groups.

Groups		Mean (standard deviation)
(JW)	Jeltrate + distilled water	99.2 (0.08) b
(JCHX)	Jeltrate + 0.2% CHX	100.0 (0.15) a
(JAgNP_0.2%)	Jeltrate + 0.2% AgNP	99.5 (0.09) b
(JAgNP_1%)	Jeltrate + 1% AgNP	99.4 (0.09) b
(AW)	Avagel + distilled water	99.6 (0.06) b

Note: CHX - 0.2% chlorhexidine digluconate; AgNPs - silver nanoparticles; JW - commercial negative control; and AW - commercial positive control. Materials composition (as provided by the manufacturers): Jeltrate: potassium alginate, calcium sulfate, tetrasodium pyrophosphate, potassium fluotitanate, polypropylene glycol, magnesium oxide, diatomaceous earth, pigments, flavoring. Avagel: potassium alginate, calcium sulfate, tetrasodium pyrophosphate, potassium fluotitanate, polyethylene glycol, magnesium oxide, diatomaceous earth, flavoring, chlorhexidine, anhydrous alcohol, and phenolphthalein.

### Surface detail reproduction

All evaluated groups successfully reproduced the 50 μm line, regardless of the solution used for irreversible hydrocolloid manipulation.

### Microbiological analysis

**Figure 2:**
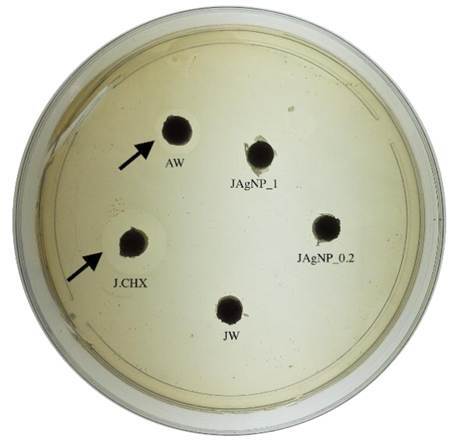
Zone of inhibition (black arrow) formed against S. mutans. JW - Jeltrate + distilled water; JCHX - Jeltrate + 0.2% chlorhexidine digluconate; JAgNP_0.2% - Jeltrate + 0.2% silver nanoparticles; JAgNP_1% - Jeltrate + 1% silver nanoparticles; and AW - Avagel + distilled water.

**Figure 3:**
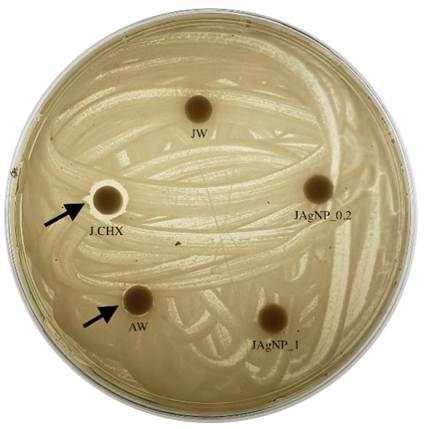
Zone of inhibition (black arrow) formed against Candida albicans. JW - Jeltrate + distilled water; JCHX - Jeltrate + 0.2% chlorhexidine digluconate; JAgNP_0.2% - Jeltrate + 0.2% silver nanoparticles; JAgNP_1% - Jeltrate + 1% silver nanoparticles; and AW - Avagel + distilled water.

Only the JCHX and AW exhibited zones of inhibition for both tested microorganisms (Figures 2 and 3). The largest zone of inhibition was observed with JCHX against *S. mutans*, and the smallest was observed with AW against *C. albicans*. Table 2 shows the presence of an inhibition zone and mean inhibition zone diameters (mm) in groups JCHX (3.4 mm to *S. mutans* and 0.87 mm to *C. albicans*) and AW (0.87 mm to *S. mutans* and 0.15 mm to *C. albicans*). In contrast, no inhibition zones were observed in the JW (commercial negative control), AgNP_0.2%, and AgNP_1% groups.

**Table 2.** Mean values (standard deviation) of dimensional stability (%) for the tested groups.

Groups		Zone of inhibition	Mean inhibition zone (mm)	
			*S. mutans*	*C. albicans*
(JW)	Jeltrate + distilled water	No	0	0
(JCHX)	Jeltrate + 0.2% CHX	Yes	3.4	0.42
(JAgNP_0.2%)	Jeltrate + 0.2% AgNP	No	0	0
(JAgNP_1%)	Jeltrate + 1% AgNP	No	0	0
(AW)	Avagel + distilled water	Yes	0.87	0.15

Note: CHX - 0.2% chlorhexidine digluconate; AgNP - silver nanoparticles; JW - commercial negative control; and AW - commercial positive control. Materials composition (as provided by the manufacturers): Jeltrate: potassium alginate, calcium sulfate, tetrasodium pyrophosphate, potassium fluotitanate, polypropylene glycol, magnesium oxide, diatomaceous earth, pigments, flavoring. Avagel: potassium alginate, calcium sulfate, tetrasodium pyrophosphate, potassium fluotitanate, polyethylene glycol, magnesium oxide, diatomaceous earth, flavoring, chlorhexidine, anhydrous alcohol, and phenolphthalein.

## Discussion

The results showed that adding an AgNP solution to the alginate at either concentration did not inhibit microbial growth. However, surface detail reproduction and dimensional stability of the molds remained unchanged in all tested solutions. Therefore, all hypotheses stated in this study were confirmed. All plaster models, regardless of the solutions used, reproduced the 50 µm line, and the maximum variation in dimensional stability was 0.8 % (Jeltrate + water), in compliance with ISO 4823 (Dentistry - Elastomeric impression materials).

Alginates are irreversible hydrocolloid materials primarily composed of calcium sulfate, potassium, sodium, or ammonium alginates, sodium phosphate, and filler particles. They form when water interacts with the alginate powder, releasing calcium ions from calcium sulfate hemihydrate and forming cross-linking points. Alginates are often used in clinical practice because they are cost-effective and easy to handle compared to other impression materials. The success of clinical procedures involving molding is closely related to the material's ability to reproduce surface details and maintain dimensional stability, resulting in a plaster model that closely resembles the original structure ^24^.

Cross-contamination is frequently observed in dental clinical practice and must be mitigated to prevent the spread of microorganisms. Thus, decontamination of impression materials is crucial for controlling cross-infection. Oral microorganisms can easily adhere to impression materials after they set, and disinfection procedures should then be performed ^6^. Immersion or spraying are commonly used for disinfecting alginate molds, but these methods can only clean the surface, leading to dimensional changes in the mold ^18^. One of the key concerns with alginate models includes poor tear resistance, dimensional instability, especially after a delay in casting the stone model, and a weak ability to reproduce surface details. Therefore, the mold's precision can be directly affected by imbibition or syneresis, which can be easily prevented by immediately filling the mold with plaster ^25^. An alternative to spraying or immersion in disinfectant solution is the use of self-disinfecting materials. This approach provides better dimensional stability than the previous ones, reducing disinfection time ^26^.

The findings of this study demonstrate that mixing the material with a disinfectant solution does not compromise the dimensional stability of the molds, as it remains within the acceptable limits set by the standard (1.5 µm - ISO 4823, 2015); however, only 0.2% CHX yielded significantly larger models. In one of the tested groups, a disinfectant was present, but it was part of the Avagel alginate composition. Jeltrate + 0.2% CHX and Avagel + water were the only groups demonstrating antimicrobial activity, unlike the Jeltrate + water and the AgNP groups. Chlorhexidine is a broad-spectrum disinfectant considered the gold standard ^27^ and is known to be a safe and effective antimicrobial agent ^26^. It has bacteriostatic properties at low concentrations and bactericidal effects at higher concentrations. CHX uptake induces quick cell death by disrupting the bacterial cell wall ^27^. However, to prevent changes in mold dimensions, other antimicrobial agents, such as AgNP, can be a good alternative.

AgNPs are currently employed in medical products and equipment because they have been reported to exhibit broad-spectrum antimicrobial effects (against bacteria, fungi, protozoa, and some viruses) and low toxicity ^28^. AgNPs target multiple bacterial targets, reducing the likelihood of resistance. In contact with bacterial DNA and RNA, AgNPs bind to sulfur and phosphorus, thereby degrading associated nucleic acids ^27^. Studies have shown that AgNPs may be more effective than CHX under certain conditions. A previous study ^(^ ^27^ demonstrated significantly stronger bacteriostatic and bactericidal effects against bacteria (*Streptococcus mutans* and *oralis, Lactobacillus acidophilus* and *fermentum*) and the fungus *Candida albicans* when compared to CHX, particularly when used as a solution. Another study ^(^ ^28^ showed that disinfection effectiveness increased with higher AgNP concentrations (0.25, 0.50, and 1%), higher powder-to-water ratios, and longer handling times. In their study, AgNPs were incorporated into the powder rather than into the mixing solution, as done in the present study. Research ^(^ ^29^ also found that incorporating AgNPs into alginate powder was more effective than disinfection with 2% glutaraldehyde, using alginate mold discs in a diffusion medium, as in the present study. Both the mode of incorporation of AgNPs into alginate and particle size appear to influence antibacterial activity. Ginjupalli et al. ^(^ ^18^ assessed antimicrobial activity using AgNPs with different particle sizes (80-100, 50-80, 30-50, and 10-20 nm) incorporated into an irreversible hydrocolloid powder. They demonstrated that 80-100 nm AgNPs had the highest dose-dependent antimicrobial activity. While previous studies identified a maximal antibacterial effect for AgNPs in the ~80-100 nm size range ^18^, biosynthesis via *Trichoderma reesei* is typically associated with much smaller particles (e.g., ~5-50 nm) ^30^. When added as a solution rather than incorporated into powder and embedded in an alginate matrix, these smaller particles likely had reduced availability and diffusion. In the present study, AgNPs were synthesized biologically using *Trichoderma reesei*, a method known to produce particles with heterogeneous sizes and surface characteristics ^30^. They were added as a mixing solution rather than incorporated into the alginate powder. These factors, combined with the limited diffusion of nanoparticles through the set hydrocolloid matrix, likely reduced their availability for microbial interaction and may explain the absence of antimicrobial activity observed in the present study, despite the well-documented efficacy of AgNPs in other models.

Surface detail reproduction, as recommended by the standards, varies by impression material type. In general, the higher the precision of the material, the better its ability to reproduce surface details. Elastomeric impression materials exhibit surface detail reproduction ranging from 20 to 75 µm, depending on the specific type (ISO 4823, 2015), whereas alginates typically reproduce details of up to 50 µm (ISO 1563, 1990). Regarding dimensional stability, the standard allows variations of up to 1.5 µm (ISO 4823, 2015). Accordingly, mixing alginate with a disinfectant solution, whether or not AgNPs are present, does not appear to interfere with the material’s properties.

Some limitations of this study are noteworthy. The biogenically synthesized AgNPs were incorporated into the mixing solution without characterization of their size distribution, morphology, stability, or ion-release behavior, all of which can influence their antimicrobial performance. Additionally, no aging or storage protocols were applied, and the diffusion-based antimicrobial assay may have been limited by nanoparticle entrapment within the hydrocolloid matrix. The mode of incorporation - mixing AgNPs into the liquid phase rather than embedding them in the powder - may also have limited their interaction with microorganisms. Future studies should therefore include detailed nanoparticle characterization, ion-release analysis, aging protocols, and direct-contact antimicrobial models, as well as the evaluation of alternative incorporation strategies, to better elucidate the underlying mechanisms and optimize the antimicrobial potential of biogenically synthesized AgNPs in alginate impression materials.

In summary, Jeltrate mixed with CHX exhibited the highest dimensional stability. All groups reproduced the 50 µm line in the plaster models, regardless of the material or solution used. Jeltrate mixed with CHX and Avagel mixed with water inhibited bacterial growth. AgNPs incorporated into alginate did not alter the dimensional stability of alginate molds.
